# Innovative Approach for Interstitial Cystitis: Vaginal Pessaries Loaded Diazepam—A Preliminary Study

**DOI:** 10.1155/2013/386546

**Published:** 2013-01-20

**Authors:** P. Capra, P. Perugini, M. Bleve, P. Pavanetto, G. Musitelli, B. Rovereto, D. Porru

**Affiliations:** ^1^Department of Urology, San Matteo Hospital of Pavia, Pavia, Italy; ^2^Department of Drug Science, University of Pavia, Pavia, Italy

## Abstract

Bladder pain is a characteristic disorder of interstitial cystitis. Diazepam is well known for its antispasmodic activity in the treatment of muscular hypertonus. The aim of this work was to develop and characterize vaginal pessaries as an intravaginal delivery system of diazepam for the treatment of interstitial cystitis. In particular, the performance of two types of formulations, with and without beta-glucan, was compared. In particular, the preparation of pessaries, according to the modified Pharmacopeia protocol, the setup of the analytical method to determine diazepam, pH evaluation, dissolution profile, and photostability assay were reported. Results showed that the modified protocol permitted obtaining optimal vaginal pessaries, without air bubbles, with good consistency and handling and with good pH profiles. In order to determine the diazepam amount, calibration curves with good correlation coefficients were obtained, by the spectrophotometric method, using placebo pessaries as matrix with the addition of diazepam standard solution. This method was demonstrated sensible and accurate to determine the amount of drug in batches. Dissolution profiles showed a complete diazepam release just after 15 minutes, even if beta-glucan pessaries released drug more gradually. Finally, a possible drug photodegradation after exacerbated UV-visible exposition was evaluated.

## 1. Introduction

Interstitial cystitis (IC), also known as painful bladder syndrome, is a distressing, chronic bladder disorder of unknown cause, with symptoms of pain, pressure, or discomfort related to the bladder and usually accompanied by a frequent and urgent need to urinate day and night [1]. Pain or discomfort is the most important and debilitating symptom of this disorder. It may be experienced as discomfort or tenderness or irritation or burning sensation in the bladder, in the form of spasms in or around the bladder, or stabbing or burning vaginal pain or simply a feeling of pressure on or in the bladder or a feeling of fullness even when there is only a little urine in the bladder. IC pain can originate not only from the bladder but also from nearby muscles, nerves, and even referred pain from other parts of the body [2]. With time, most patients learn the subtle difference in symptoms between an IC flare caused by direct bladder irritation (i.e., diet) versus a muscle spasm (i.e., sex, driving in a car, etc.). Bladder wall irritation has a tendency to create more of a sharp, intense pain. Pelvic floor muscle spasms create a deeper, heavier burning sensation that can make sitting more uncomfortable. Nerve pain can be more electrical, hot, or searing in nature. Each potential source of pain has its own symptoms and treatments. 

Despite all the research and studies that have been carried out, as yet no possibility has been found of curing this disease, nor there is a single drug that is effective in all patients. Nevertheless, there are many different options to try. Treatment of IC should focus on the pain and urological symptoms such as urgency and frequency. However, pain management should play an important role [3]. If the pain is very severe and fails to respond to standard treatment, a pain clinic referral may be advisable.

Actually, for pain therapy many drug classes have been administrated. Benzodiazepines (BZDs) are considered to be the treatment of choice for acute management of severe pain. BZDs have a rapid onset of action once delivered into the central nervous system and are safe [4, 5]. Diazepam, in particular, is a long-acting benzodiazepine with anticonvulsant, anxiolytic, sedative muscle relaxant and amnesic properties. However, it has a short duration of action and should be given intravenously or rectally. In particular, the absorption after oral administration of tablets is usually slower than after parenteral or rectal administration [6]. Instead, dosage forms designed for rectal administration should not cause irritation, should show good retention in the lower region of the large intestine, and should be suitable enough to be accepted by the patient. 

According to these considerations, Balkis studied the release of diazepam from different conventional and hollow type suppository bases and he concluded that the best results were obtained using glycerol-gelatin and glycerol-PEG1540 as water soluble bases [7].

Currently, some doctors are prescribing vaginal diazepam (Valium) suppositories or tablets to help relieve the pain of pelvic floor dysfunction, interstitial cystitis, vulvar pain, and sexual pain. This causes less drowsiness as a side effect than oral valium, but nevertheless it may still produce mild sedation [8]. Dosage is usually 5–10 mg valium compounded (in a paraffin base), starting once nightly and titrating. An alternative route to rectal administration of diazepam can be considered the intravaginal route, to consider adjunctive treatment of bladder pain in interstitial cystitis. Considering all the above-mentioned facts, diazepam loaded vaginal pessaries have been formulated and characterized in order to propose an innovative therapeutic approach for painful symptoms, characteristics of interstitial cystitis [9].

In particular, diazepam loaded vaginal pessaries, described in this study, have been formulated with beta-glucan addition as active excipient. According to the literature, beta-glucan prevents and treats mucositis through wound healing and tissue reorganization [10], and this aspect is very interesting since patients affected with interstitial cystitis show a damaged mucous tissue with ulcerations. 

## 2. Materials and Reagents

Diazepam powder (DZP) was provided by FIS-SPA (Italy); *β*-glucan (CM-glucan granulate, SD = 0.85) and gelatine (mesh size 20, batch 4767) pellets were purchased, respectively, from Mibelle Biochemistry and Lapi-Gelatine s.p.a. Diazepam was obtained with official authorization. All other materials were of analytical grade.

### 2.1. Preparation of DZP Vaginal Pessaries

Vaginal loaded pessaries were prepared by hydrating gelatine in water over night. Gelatine was heated in an ultrasound thermal bath, at 85°C, until complete fusion. At the same time glycerol and *β*-glucan aqueous solution have been separately heated (at 85°C) and finally mixed together. *β*-glucan-glycerol mixture was added to fused gelatine and vigorously stirred in order to obtain a homogeneous formulation. Finally, DZP powder was added, and the formulation obtained was sonicated and cast in the moulds. Placebo pessaries have been also prepared with the same procedure without DZP addiction. Placebo and loaded pessaries were prepared according to European Pharmacopeia (7th edition). All batches, placebo and loaded, were stored at 4°C, and they were prepared in triplicate.

In Table 1 batches composition was reported.

### 2.2. Drug Content in Vaginal Pessaries

DZP content was determined using spectrophotometric technique by external standard method. Analysis was performed by UV-visible spectrophotometer (Spectrophotometer UV-Vis Agilent 8453). Calibration curve was obtained by considering placebo samples and five different scheduled concentrations of DZP (6.40–87.06 *μ*g/mL).

Loaded pessary (3.6 g) was transferred to vial and 4 mL of absolute ethanol was added, and the vial was placed in the ultrasonic bath at 37°C until complete softening. The samples were stirred at 600 rpm for 15 minutes in order to allow gelatine precipitation, and then 1 mL of the suspension, transferred to a microtube, has been centrifuged at 14.000 rpm, at 24°C for 10 minutes in order to assure gelatine separation to the microtubes bottom.

Colorimetric assay was performed by adding 0.5 mL of supernatant, 1 mL of 3,5-dinitrobenzoic acid, and 0.5 mL of 7 M sodium hydroxide [11]. The absorbance of the sample was measured at 530 nm.

### 2.3. Evaluation of DZP Distribution into Loaded Formulations

In order to evaluate drug homogenous distribution into pessaries, different parts of each sample were collected: base, body, and summit. In particular, 1.2 g of each part was added to absolute ethanol. Spectrophotometric analysis was determined by the experimental protocol described above. 

### 2.4. Evaluation of pH of Vaginal Formulations

All samples, loaded and placebo, were soaked in 2 mL of phosphate buffer solution, pH 4.2 at 37°C. After scheduled times (15, 60 minutes and 4, 6, 24 hours) pH measurements were carried out in order to evaluate the possible changing of pH incubation medium. 

### 2.5. *In Vitro* Release Study

**Figure 1:**
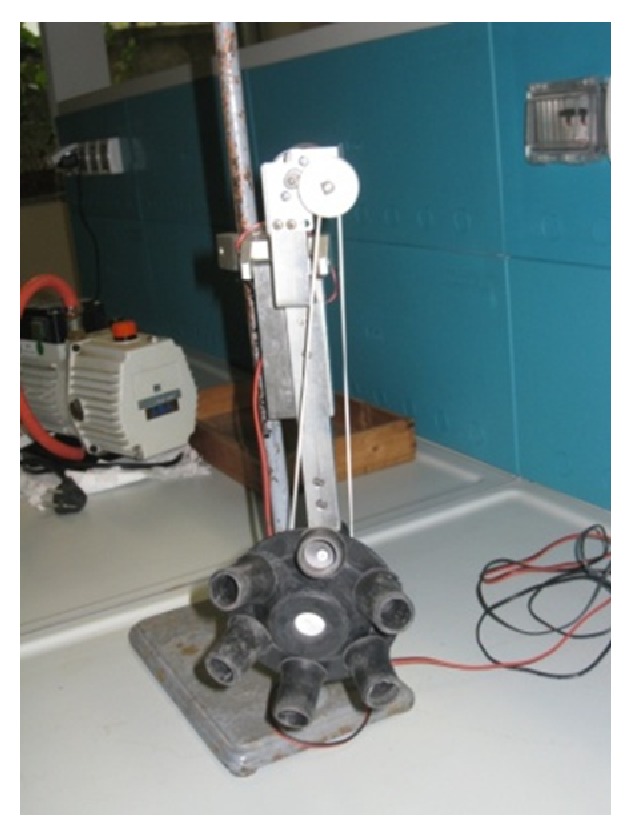
*In vitro* release apparatus: instrument at rotational motion in order to simulate mechanical stress in a vaginal environmental.

In our laboratory, a modified *in vitro* test apparatus was suitably realized (Figure 1) in order to simulate intravaginal conditions and to discriminate formulation performance. For this purpose, vials were allocated to suitable designed rotor in order to guarantee planetarium movement. In particular, vials were fixed to conical rotor with a well-defined angle. The experiment was set up in a water bath at 37 ± 1°C.

Loaded pessaries (3.6 g) were poured in a glass vial in the presence of phosphate buffer pH 4.2 (2 mL) exposed to speed at 37 rpm. At scheduled times (2, 5, 7, 15, 25, and 35 minutes) liquid phase was withdrawn and stirred for few minutes in order to guarantee complete dispersion of diazepam. Drug release analyses were carried out by the spectrophotometric method reported above. In particular, 1 mL of absolute ethanol was added to 0.5 mL of liquid sample. After stirring and centrifugation, 0.5 mL of the supernatant was withdrawn to perform colorimetric assay. Withdrawn aliquots were analyzed spectrophotometrically at 530 nm (Spectrophotometer UV-Vis Agilent 8453). All the experiments were carried out in triplicate, and average values were presented. 

### 2.6. Photostability Study

Diazepam, according to European Pharmacopoeia 7th Edition, is photosensible. For this reason, pessaries were submitted to artificial solar light by Suntest XLS +II (Atlas).

The assay was carried out in order to evaluate the possible interaction between packaging and formulation. Samples were maintained in mould (primary packaging) or in glass (manufacturing packaging). 

At the end of the study samples in Suntest were compared with standards stored at room temperature.

The instrument was set up according to standard European procedure [12] and precisely to the following parameters:

 time: 4 hours corresponding to 192 hours solar light,
 irradiation control: 300–800 nm,
 irradiation W/m^2^: 750,
 room temperature: 35°C,
 black standard temperature (BST): 45°C.

## 3. Results and Discussion

Diazepam is a long-active benzodiazepine with several properties, such as anticonvulsant, anxiolytic, sedative muscle relaxant, and amnesic, but it is also administered in the treatment of insomnia, febrile convulsions, status epilepticus, and alcohol withdrawal symptoms. It could be administered via different routes: orally, IV injections, rectal solutions, rectal gels, and suppositories. However, oral and IV route, although they constitute a simple and rapid way of administration, the first shows erratic and slower absorption, the second, instead, is bound by the presence of medical personnel. On the basis of these assumptions and of the awareness that a topical formulation, such as suppositories and vaginal pessaries, could have a prompt absorption and rapid entry to the central nervous system, an expert team of urologists have proposed vaginal pessaries loaded diazepam, as practical, efficacious, and lacking side effects administration form in the treatment of interstitial cystitis. However, a lower dose of drug has been considered because local bioethical committee highlighted the requirement, in a clinical I phase, to begin with a low dose in order to provide for haematic drug titration, evaluating and monitoring its clinical and side effects.

According to Pharmacopoeia, two different formulations of pessaries with and without beta-glucan were considered and set up. Beta-glucan is a polysaccharide with significant therapeutical profile. Antioxidant, anti-inflammatory, and wound healing properties have been attributed to this polysaccharide, and this represents scientific rational about its use in vaginal pessaries formulation. 

Interstitial cystitis in fact is characterized by alterations of bladder wall with reddenings and ulcerations that beta-glucan could be able to heal. However, beta-glucan contributes also to give more consistence to formulation. 

Numerous experimental parameters allow to obtain standardized and reproducible vaginal formulations: hydration temperature and time of gelatin, sonication time, manufacturing temperature, diazepam addition, and pessary storage. The setup of prepared formulations has been carried out according to the process conditions reported in Table 2.

Vaginal systems, here proposed, although simple to reproduce, were characterized from a matrix more and more complex: a small amount of diazepam is suspended in a mixture of gelatin, water, glycerin, or/and beta-glucan. Consequently, formulator must deal with two critical aspects: drug is not homogeneously distributed into matrix, due to its moderate solubility into base matrix. The second aspect is that it is difficult to determine the exact amount of active present into formulation. 

**Figure 2:**
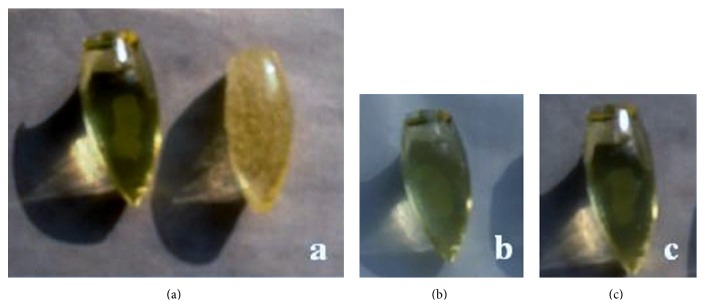
Vaginal pessaries: (a) comparison between pessaries obtained with sonication (on the left) and pessaries obtained without sonication (on the right); (b) loaded pessary obtained with glycerin, water, and gelatin; (c) loaded pessary obtained with glycerin, water, gelatin, and beta-glucan.

In Figure 2, pessaries, obtained with and without addition of beta-glucan, were reported. Images show how different formulations could seem apparently similar due to gelatin presence which gives a characteristic transparent and yellow color to the batches. However, it is possible to observe, in the second batch, several air bubbles.

Finally, it is important to underline that for all batches a water loss, correlated to temperature storage, has been registered. A water vapor exchange between matrix and environment was hypothesized.

### 3.1. Drug Content in Vaginal Pessaries

In order to evaluate diazepam loaded into pessaries, a spectrophotometric method was set up. The method proposed for active determination in the tablets and ampoules was suitably adapted to vaginal formulations. According to this colorimetric method, diazepam yields intensely red colored product when it reacts with 3,5-dinitrobenzoic acid in alkaline medium. The red complex is called Meisenheimer complex, and the reaction is called Janovsky reaction [11].

**Figure 3:**
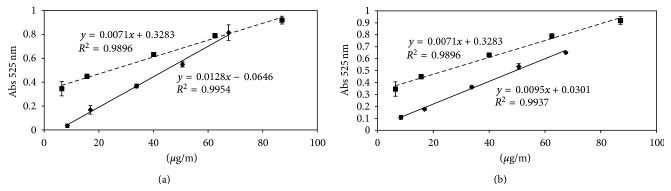
Calibration curves: (a) (- - -) calibration curve obtained with standard solution of diazepam and (—) calibration curve with standard in association with gelatin placebo batches. (b) (- - -) Calibration curve obtained with standard solution of diazepam and (—) calibration curve with standard in association with beta-glucan placebo batches.

Three different calibration curves were fitted. In Figures 3(a) and 3(b) standard diazepam solutions and standard solution more placebo batches (A batches or B batches) have been compared. By comparing curves it is possible to observe that the matrix, made of gelatin, beta-glucan, and glycerin, is able to interfere with diazepam determination. In fact in both figures, curve profiles are not parallel but they tend to cross. Moreover, curves, with standard additions to pessaries, show correlation coefficients above, respectively, 0.9954 and 0.9937. 

Calibration curves are applied to determine diazepam loading: Table 3 reports drug percentage of gelatin and beta-glucan batches, showing, respectively, over and underestimation of drug. In the first case, water and excipient loss, having reference to manufacturing and storage phase, explain a higher percentage of diazepam. On the other hand, the lower in beta-glucan samples could be explained by hypothesizing that polysaccharide structure binds drug. 

Diazepam is a water-insoluble drug, and its incorporation into vaginal formulation is dependent on the preparation process. In order to attain optimal diazepam loading, several factors, such as excipients and process parameters, were considered. In particular, it was observed that 2% of beta-glucan increases the viscosity of fused mass, and consequently it guarantees a homogenous distribution of diazepam powder. As it is reported in Table 3, in fact, gelatin pessaries show a higher diazepam percentage on their bottom due to drug precipitation tendency. This effect could be related to low viscosity of this formulation type.

After drug addition, sonication is one of the main process parameters that contributes to avoid drug aggregates formation and to assure a good drug distribution.

### 3.2. pH Evaluation of Vaginal Formulations

According to many researchers and clinicians, neural modulation and transmission of pain in IC are accentuated by direct stimulation in low pH environments. Indeed, over the past 20 years or more, alkalinizing agents in managing cystitis-like symptoms have been used with good results. However, recently, several studies [13, 14] reevaluated the relationship between urinary pH and cystitis symptoms, concluding that there was no relationship between them for a ranging between pH 5.0–8.0. Thus, in the light of the previous scientific discussions, it is extremely important, in preclinical phase, to determine pH changes of topical formulation during their disaggregation.

**Figure 4:**
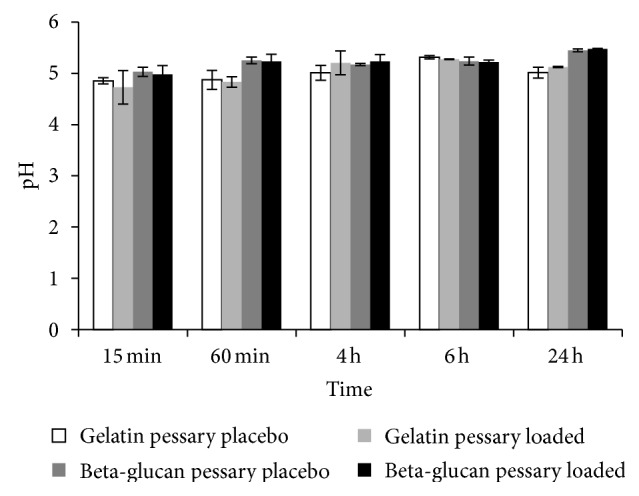
pH evaluation of pessary batches at scheduled times, incubated in phosphate buffer 4.2.

In Figure 4 pH values of pessaries incubation medium are reported. From the figures it is evident that pH of all formulations maintains, just after 15 minutes, values to ~5. These weakly acid values are attributed to gelatin, obtained from alkaline hydrolysis. Indeed, the same figures show a constant profile until 24 hours.

### 3.3. *In Vitro* Release Study of Diazepam from Pessaries

*In vitro* release study was carried out using phosphate buffer pH 4.2 as preliminary medium of dissolution in order to simulate physiological pH of vaginal environment.

**Figure 5:**
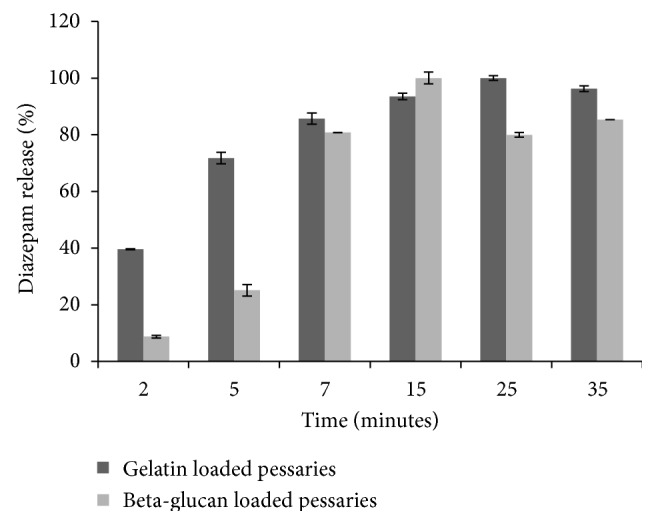
Diazepam cumulative release profiles in buffer phosphate pH 4.2.

Release profiles of diazepam from vaginal pessaries are shown in Figure 5. As it is possible to observe from the figures, beta-glucan and gelatin batches release completely the drug at 15 and 25 minutes, respectively. In particular, gelatin pessaries release about 40% diazepam just after 2 minutes against about 8% of beta-glucan samples. Figure 5 demonstrates also that, probably due to the mechanism of drug inclusion into polysaccharide network, beta-glucan permits a gradual release of the drug from matrix according to the previous discussion. Moreover, after plateau achievement, gelatin batches maintain high profile (~100%), while beta-glucan ones show values decrease (80%), probably due to an assumed drug-polysaccharide interaction.

Drug release profiles are agreed with previously disaggregation test (data not reported). In fact, beta-glucan pessaries disaggregate completely at 30 minutes, 10 minutes more than gelatin ones.

### 3.4. Photostability Study

Photosensitivity, associated with benzodiazepines, has recently been reported [15, 16]. The ICH Harmonized Tripartite Guideline 10 and recent FDA draft guidance [12] state that light testing should be an integral part of stress test. Therefore, it is of interest to test the stability of diazepam photochemical stress conditions. Gallardo et al. [17] observed that, after light exposition, degradation products formed. On the basis of these considerations all vaginal formulations have been submitted to UV-visible irradiation in order to verify the screening effect of mould onto pessary and, so, on drug.

As it is possible to observe in Table 4, independently from batch type, all samples submitted to accelerated conditions and stored in mould, they show a decrease of drug amount due to degradation power of irradiations on diazepam, but also, due to a possible diazepam-packaging interaction. In Pharmacopeia it is reported that polyvinyl chloride, when being a constituent of infusion packaging, could interact with diazepam and consequently adsorb it. 

Finally, it is interesting to underline that beta-glucan samples have maintained their structure and form at the end of the assay, instead, gelatin pessaries appear completely fused, probably due to the lack of texture agent, as beta-glucan. This confirms that polysaccharide guarantees a consistence and a slow release of drug from formulation, just observed above.

## 4. Conclusions

In this study, an interesting water-insoluble drug for bladder pain cure in interstitial cystitis therapy has been loaded in a topical formulation, simple to administrate and without side effects onto central system.

In alternative to classical formula reported to official Pharmacopeia, this work suggests vaginal pessaries with addition of polysaccharide with notable therapeutic and technological properties. The choice of polysaccharide addition seems to contribute successfully to achievement of handling pessaries with good organoleptic characteristics, when the drug is homogeneously distributed in a matrix. 

Moreover, drug release test apparatus, designed in our laboratories, permits to simulate mechanical stress in a vaginal environmental. In particular, this apparatus presents different advantages with respect to official test. First of all the volume of dissolution medium required and the system geometry assure *in vivo* conditions. Furthermore, in these conditions, even extremely low drug amount can be easily quantified.

Despite that dissolution medium volume is small but coherent with the literature, pessaries dissolved and released completely the drug within 30 minutes, guaranteeing probably a rapid pharmacological efficacy samples alteration in terms of organoleptic properties and pharmacological efficacy.

The promising results obtained in this study represent the opportunity to develop a clinical study, when it is possible to evaluate its therapeutic efficacy on the interstitial cystitis patients. 

## Figures and Tables

**Table 1:** Diazepam loaded pessaries and placebo pessaries composition.

Batch	Glycerol	Water	Gelatine	*β*-glucan	Diazepam
A	50%	40%	10%	—	—
B	50%	39.94%	10%	—	0.06%
C	50%	38%	10%	2%	—
D	50%	37.94%	10%	2%	0.06%

**Table 2:** Parameters of formulation process in manufacturing and storage phase.

Set-up phase	Parameters evaluation
	Temperature of about 75°C guarantees a complete fusion of all excipients and absence of aggregates
Manufacturing	Sonication avoids air bubbles formation (Figure 2(a)) contributing to obtain a homogenous dispersion of diazepam powder into fused matrix
	Mould filling has been performed by analytical balance in order to uniform mass content at about 3.6 grams
Storage	Pessaries must not be exposed to solar light and they must be stored at 4°C in order to preserve diazepam activity

**Table 3:** Loaded efficiency (%) and distribution evaluation of diazepam in gelatin and beta-glucan batches.

	Loaded diazepam percentage	Content uniformity (%)		
		Top	Central	Bottom
Gelatin batch	110.43% ± 4.47	32%	31%	37%
Beta-glucan batch	81.23% ± 3.49	33%	33%	34%

**Table 4:** Photodegradation of diazepam after UV ray exposition: percentage amount of drug in batches stored in mould and in batches stored in manufacturing container (borosilicate glass).

		Standard condition	Accelerated conditions
Gelatin sample	Formulation in polymeric mould	121.94% ± 0.003	99.13% ± 0.067
	Formulation in glass (standard sample)	93.75% ± 0.002	85.09% ± 0.006

Beta-glucan sample	Formulation in polymeric mould	105.65% ± 0.052	82.36% ± 0.013
	Formulation in glass (standard sample)	100.53% ± 0.019	76.14% ± 0.021
